# Actinic keratoses in subjects from la Mitad del Mundo, Ecuador

**DOI:** 10.1186/s12895-020-00109-8

**Published:** 2020-10-27

**Authors:** Martha Fors, Paloma González, Carmen Viada, Kirsten Falcón, Santiago Palacios

**Affiliations:** 1 Universidad de Las Américas (UDLA), Redondel del Ciclista, Antigua Vía a Nayón, Quito, Ecuador; 2 Universidad de Las Américas, Avenida de los Granados y Vía a Nayón, Quito, Pichincha Ecuador; 3 Centro de Inmunología Molecular, Habana, Cuba; 4 Pontificia Universidad del Ecuador (PUCE), Quito, Ecuador

**Keywords:** Actinic keratoses (AKs), Cross-sectional study, Epidemiology, Risk factors

## Abstract

**Background:**

Quito, the capital of Ecuador due to its geographical location, has a high skin cancer incidence. Actinic keratoses, as premalignant lesions, are precursors of nonmelanoma skin cancer, and the prevalence of this medical condition in the country is unknown.

**Methods:**

An observational, cross-sectional study was performed to assess the prevalence of actinic keratoses (AKs) in a rural area of Quito. Visual skin exams, dermoscopy and biopsy of suspicious lesions were performed.

**Results:**

A total of 254 subjects older than 40 years old (71.3% female) were enrolled. The general AK prevalence was 22.4%; in women, the prevalence was 23.6%, while in men, it was 19.4%. The prevalence rates of basocellular and squamous cell carcinomas and Bowen disease were 1.6, 0.8 and 0.4%, respectively. No statistical associations were found between AKs and the studied variables.

**Conclusions:**

This study was the first reporting the prevalence of premalignant lesions in Ecuador. We could not demonstrate a relationship between the presence of AKs and any of the known risk factors for their development.

## Background

Nonmelanoma skin cancer (NMSC) includes cutaneous squamous cell carcinomas and basal cell carcinomas. The term NMSC practically refers to keratinocyte carcinoma, basal cell carcinoma (BCC) and squamous cell carcinoma (SCC), and they account for 99% of the tumors in this group. Actinic keratoses (AKs) are considered premalignant lesions [1], and they are a common keratinocytic intraepidermal neoplasia often occurring on the chronically sun-exposed skin of Caucasian individuals [2, 3].

The worldwide incidence of nonmelanoma skin cancer (BCC, SCC, and Bowen’s disease) is very high [4, 5]. AKs can progress to SCC in approximately 0.1–20% of the lesions [6, 7], and they are of public health importance since their presence has been associated with a significantly increased incidence of non-melanoma skin cancers.

Occupational exposure to ultraviolet (UV) radiation is high in many outdoor occupations; recent studies have suggested that persons working in such occupations are more likely to develop nonmelanoma skin cancer [8]. Geographically, Ecuador is located between 2° North latitude and 5° South latitude at 2850 m (9350 ft), which is a very high altitude. For every 1000 m, UV radiation levels increase in a range from 10 to 12% [9].

According to the National Tumor Registry System of Ecuador, the incidence of nonmelanoma cancer in the period of 1986–1990 was 21 per 100,000 inhabitants, and in the period of 2006–2010, it was 33 per 100,000, indicating an increase of 57% [10].

Excessive exposure to UV radiation is very frequent in our country. Limited data are available on the prevalence and risk factors for AKs in the Ecuadorean population, and because of the tendency of premalignant lesions to convert to malignancies, the identification of patients with AKs is very important to establish the basis of screening programs to support public health policies.

The aim of this study was to prospectively evaluate subjects living in a rural area of Quito to assess the prevalence of premalignant skin lesions (AKs) and skin cancer (NMSC) in this population. We also aimed to evaluate the relationships of AKs with age, sex, ethnicity, education level, alcohol consumption, smoking history and sun exposure habits. Skin phototype, photoaging and skin damage were also studied.

## Methods

### Study design

This study was a cross-sectional study to assess the prevalence of AKs.

### Sample size

We included 254 Ecuadorian adults in this study. The subjects were chosen by simple random sampling from lists provided by the authorities of each town that we visited (seven towns located in la Ruta Escondida de la Mitad del Mundo).

### Study participants

Full-body skin examinations were performed among participants aged 40 years old or older. The diagnosis of NMSC was made clinically and with dermoscopy as an aid for diagnosis. Skin biopsy, in addition to confirmation of diagnosis, was performed for all suspected lesions, allowing for their classification. Patients with these lesions were referred to health institutions of the Ministry of Public Health or to Centro de la Piel (CEPI) in the city of Quito.

### Variables

Age, sex, ethnicity, education, alcohol consumption, smoking history and sun exposure habits were recorded. For the analysis, age was dichotomized into ages 40–65 and 65 and older. Educational level was classified into six categories: none, primary, secondary, high school, technical and university qualifications. Smoking status was classified into three categories: nonsmoker, ex-smoker and smoker. Other variables were also grouped. Skin phototype was measured with the Fitzpatrick Skin Phototype Classification (FSPC, type I, II, III, IV, V or VI). Severity grading of photoaging was performed using the Glogau scale (type I “no wrinkles” to type IV “only wrinkles”). Skin damage was assessed using the SCINEXA scale.

### Statistical analysis

Quantitative variables are expressed as the means and standard deviation. Absolute numbers and percentages were used for qualitative variables. The prevalence of actinic keratoses was calculated using as the denominator the total number of subjects in the sample and as the numerator those subjects with AKs.

The Chi square-test or Fisher’s exact test was used for intergroup comparisons as appropriate. Multivariate analysis was used for a logistic regression model. Variables were entered into the logistic model if the *p* values were less than 0.25 in the univariate analysis. Statistical tests were two sided, with an alpha of 0.05. SPSS 24.0 for Windows was used for data processing.

## Results

**Fig. 1:**
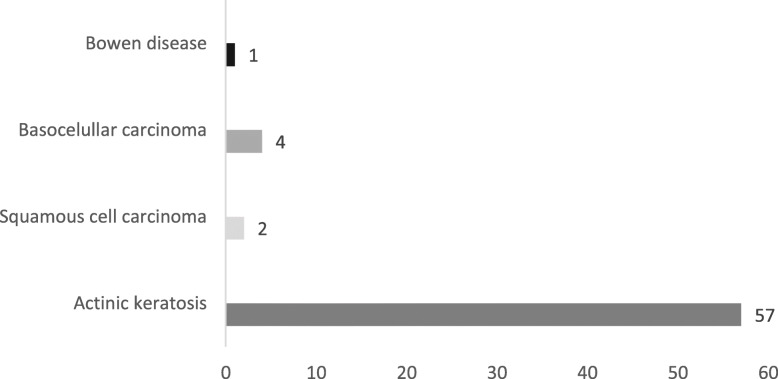
Number of subjects with nonmelanoma skin cancers

The study included 254 subjects living in La Ruta Escondida de la Mitad del Mundo. The prevalence rates of basocellular and squamous cell carcinomas and Bowen disease were 1.6, 0.8 and 0.4%, respectively. NMSC was diagnosed by biopsy. Actinic keratoses were diagnosed in 22.4% (95% CI: 17–28) of the included subjects (Fig. 1).

Women comprised the majority (71.3%) of the sample. The prevalence according to sex was 23.6% for women (43 of 182), while the frequency of this medical condition in men was 19.4% (14 of 72).

The participants’ mean age was 60.8 ± 13.8 years old, while the median age was 60 years old.

Most of the participants were between 40 and 65 years old and had less than a complete primary education. Almost 90% of the included participants were Mestizos and had no drinking history. Actinic keratoses were more frequent in subjects older than 65 years old, as well as in Mestizos. No relationship was established between the studied variables and the presence of actinic keratoses (Table 1).

**Table 1:** Characterization of participants according the presence of actinic keratoses

Demographics	Actinic Keratoses				*P* value
	No *n* = 197 (77.6%)		Yes *n* = 57 (22.4%)		
	No.	%	No.	%	
**Sex**					
Male	58	80.6	14	19.4	0.47
Female	139	76.4	43	23.6	
**Age group**					
40–65	127	80.4	31	19.6	0.21
> 65	70	72.9	26	27.1	
**Education**					
None	19	65.5	10	34.5	0.16
Primary	111	75.5	36	24.5	
Secondary	38	86.4	6	13.6	
High school	5	100.0	0	0.0	
Technical	4	100.0	0	0.0	
University	20	80.0	5	20.0	
**Ethnic**					
Mestiza	173	77.4	50	22.6	0.98
Other	24	77.6	7	22.4	
**Alcohol consumption**					
Never	116	78.4	32	21.6	0.31
Hardly ever	75	77.3	22	22.7	
Often	6	75	2	25.0	
Always	0	0	1	100.0	
**Smoking history**					
Nonsmoker	19	79.2	5	20.8	0.50
Ex-smoker	158	76.3	49	23.7	
Smoker	20	87%	3	13.0	

Most of the subjects included in this study had no sun protection. We found that 35 subjects of the 57 with AKs (61.4%) did not use protection against the sun. Most subjects with AKs worked outdoors, and those with more than 6 h outdoors were more frequent. More than 70% of the subjects with actinic keratoses never used makeup. The relationships between AKs and all sun exposure habits were not statistically significant (Table 2).

**Table 2:** Sun exposure and protection habits according to the presence of actinic keratoses

Habits	Actinic Keratoses				*P* value
	No *n* = 197 (77.6%)		Yes *n* = 57 (22.4%)		
	No.	%	No.	%	
**Sun protection behavior**					
Yes	69	75.8	22	24.2	0.62
No	128	78.5	35	21.5	
**Sun exposure per day**					
Less than 3 h	75	79.8	19	20.2	0.40
From 3 to 6 h	39	70.9	16	29.1	
More than 6 h	83	79.0	22	21.0	
**Frequency of sun protection**					
Never	126	78.3	35	21.7	0.70
Hardly ever	31	73.8	11	26.2	
Often	15	71.4	6	28.6	
Always	25	83.3	5	16.7	
**Makeup use**					
Never	160	79.2	42	20.8	0.13
Hardly ever	25	65.8	13	34.2	
Often	5	71.4	2	28.6	
Always	7	100.0	0	0.0	
**Working outdoors**					
Yes	153	77.7	44	22.3	0.90
No	44	77.2	13	22.9	

More than 60% of subjects with AKs had mild and moderate elastosis. Cutis rhomboidal was also more frequent in subjects with AKs. There were nonsignificant differences between the groups (Table 3).

**Table 3:** Prevalence of UV light-related signs of skin aging according actinic keratoses

	Actinic Keratoses				*P* value
	No *n* = 197 (77.6%)		Yes *n* = 57 (22.4%)		
	No.	%	No.	%	
**Severity of elastosis**					
None	58	29.4	18	31.6	0.76
Mild	92	46.7	23	40.4	
Moderate	41	20.8	13	**22.8**	
Severe	6	3.0	3	5.3	
**Cutis rhomboidal**					
Yes	34	17.3	12	**21.1**	0.51
No	163	82.7	45	78.9	
**Favre-Racouchot**					
Yes	1	0.5	2	3.5	0.06
No	196	99.5	55	96.5	

Most of the subjects were classified as type II or III on the Glogau scale (moderate or advanced photoaging). In patients with AKs, more than 65% had Fitzpatrick skin type IV, and 24.6% of patients had skin type III. The distribution of patients with AKs among all of the variables included was not statistically significant (Table 4).

**Table 4:** Prevalence of photoaging, skin phototype and skin damage according actinic keratoses

Scales	Actinic Keratoses				***P*** value
	No ***n*** = 197 (77.6%)		Yes ***n*** = 57 (22.4%)		
	No.	%	No.	%	
**Glogau**					
I	16	8.1	3	5.3	0.29
II	66	33.5	22	**38.6**	
III	86	43.7	19	33.3	
IV	29	14.7	13	22.8	
**Fitzpatrick**					
I	0	0	0	0	0.62
II	5	2.5	1	1.8	
III	69	5.0	15	**24.6**	
IV	114	57.9	38	**66.7**	
V	9	4.6	3	**5.3**	
VI	0	0	0	0	
**SCINEXA (skin damage)**					
Yes	154	78.2	45	78.9	0.90
No	43	21.8	12	21.1	

Table 5 shows the unadjusted and adjusted associations between the presence of AKs and possible risk factors. None of these factors represented a risk for the development of AKs in this population.

**Table 5:** Unadjusted and adjusted associations between AKs and some possible risk factors

Characteristics	Nonadjusted	Adjusted
	OR (95% CI)	OR (95% CI)
**Sex**		
Male	Reference	
Female	1.28 (0.65–2.52)	1.50 (0.68–3.30)
**Age**		
40–65	Reference	
> 65	1.57 (0.83–2.76)	1.71 (0.76–3.84)
**Ethnicity**		
Other	Reference	
Mestiza	0.99 (0.40–2.43)	0.84 (0.31–2.26)
**Fitzpatrick**		
II	Reference	
III	1.02 (0.11–9.50)	1.20 (0.12–0.57)
IV	1.73 (0.19–15.33)	2.15 (0.23–20.12)
V	1.11 (0.07–15.53)	1.17 (0.08–17.07)
**Working outdoors**		
No	Reference	
Yes	1.02 (0.50–2.07)	1.22 (0.52–2.85)
**Glogau**		
I	Reference	
II	1.77 (0.47–6.68)	1.63 (0.42–6.28)
III	1.17 (0.31–4.45)	0.93 (0.23–3.75)
IV	2.39 (0.59–9.65)	1.44 (0.29–7.12)

## Discussion

To our knowledge, there have been no previous studies of AK prevalence in our country. This medical condition is considered a precancerous epidermal lesion caused by long-term exposure to UV radiation, which can progress to squamous cell carcinoma. Bowen’s disease is also a representative cutaneous premalignant lesion.

Many epidemiological studies have examined the prevalence of malignant cutaneous tumors, but epidemiological studies of premalignant lesions have not been very frequent. Previous studies among Caucasian populations have provided substantial epidemiological data, but little has been published about trends for skin cancers in our population, which has a darker skin color.

This study included age, sex, ethnic group, alcohol consumption, smoking history and sun behavior/exposure habits. The Glogau scale for photoaging, Fitzpatrick scale and SCINEXA scale were used to evaluate skin conditions to assess for possible risk factors for the development of actinic keratoses. AKs are a chronic disease arising on skin with actinic damage, requiring treatments, as well as regular follow-up; therefore, an opportune diagnosis and the control of possible causes are necessary.

AKs were the most frequent premalignant lesions. In our study, 24.2% of all of the subjects included in the study presented this kind of lesion. Bowen’s disease appeared in 1.6% of the included subjects. AK prevalence was higher in patients > 65 years of age and in patients with Fitzpatrick skin types II and III. These results are similar to those obtained by Akdeniz et al. and Hahnel et al. [11, 12]. Similar figures of AK prevalence were found in the EPIQA study by Ferrandiz et al. [13].

Eder et al. [7] showed an overall AK prevalence of 31.0%, which was higher in men than in women. Similar results were found by Dziunycz et al. [14]; nevertheless, the current study reported that AKs were more frequent in women, but we also had more women than men in our sample.

Many studies [15, 16] have demonstrated a high prevalence of AKs among outdoor workers, and in the current study, the number of subjects with this type of lesion who work outdoors was also high. The study region is mainly devoted to growing flowers and other agricultural tasks, and most of the participants spend long periods of time outdoors, usually without adequate protection from the sun. Incomes are also very low, and buying sunscreen is not an option for this population. Nevertheless, they use other ways to protect themselves from the sun, such as wearing hats and long sleeves.

The pattern of continuous sun exposure is protective in people who have good adaptability, and it is our belief that the subjects of this study are well adapted.

Although it is known that there are several risk factors for AK development, such as sex, age, UV exposure and skin type, this study could not support this theory since we did not find any statistical relationships among possible risk factors and the presence of AKs or other premalignant lesions.

The role of UV exposure in the development of AKs has been discussed by some authors [17–19], and only in a few studies has a negative or no relationship between sun exposure and the sun-related risks associated with the occurrence of AKs and/or skin cancer been reported [20].

Multiple genetic and environmental factors might interfere with the risk of developing AKs [21]; in particular, in this study, we could not identify these factors. The relative risk of AKs was 14.1 times higher in fair skinned patients than in those with dark skin [22].

According to a study of Japanese subjects, the intensity of solar radiation is directly related to the prevalence of AKs; three times more lesions were observed in patients living in lower latitudes who were exposed to double the incidence of ultraviolet radiation B (UVRB) [23]. The subjects from this study live in such areas, so further studies should be performed to compare the prevalence of AKs with other subjects living in different regions of Ecuador.

## Conclusions

In our consideration, our results provide a reliable estimate of the prevalence of AKs in this region. This study was the first reporting premalignant lesion prevalence in Ecuador since AKs are the most common diagnosis and finding not related to any of the known risk factors for their development.

### Limitations

It is well known that UV exposure plays an important role in the development of AKs and NMSC, but the intensity and duration of UV exposure were not measured in the current study, constituting one of the most important limitations of the study.

Similarly, we have not evaluated the intensity and duration of the smoking history, which might be another important modifiable risk factor. Another limitation of the study includes the lack of information on past AKs and timing of first appearance before skin examination performed during this study. Most of the data were based on patient self-reports. Most of the subjects who were recruited were women.

## Data Availability

The datasets used and/or analyzed during the current study are available from the corresponding author upon reasonable request.

## Abbreviations

AKs: Actinic keratoses
CEISH-UDLA: Ethics committee from the university UDLA
SCINEXA: SCore for INtrinsic and EXtrinsic skin Aging
UDLA: Universidad de Las Américas
UVRB: Ultraviolet radiation B
UV: Ultraviolet
